# Stereotactic body radiotherapy for very elderly patients (age, greater than or equal to 85 years) with stage I non-small cell lung cancer

**DOI:** 10.1186/1748-717X-9-138

**Published:** 2014-06-16

**Authors:** Shinya Hayashi, Hidekazu Tanaka, Yuuichi Kajiura, Yasushi Ohno, Hiroaki Hoshi

**Affiliations:** 1 Department of Radiology, Gifu University Graduate School of Medicine, Yanagido 1-1, Gifu 501-1194, Japan; 2 Department of Radiology, Gifu University Hospital, Yanagido 1-1, Gifu 501-1194, Japan; 3 Department of Radiology, Chiyuno kosei Hospital, Wakakusadoori 5-1, Seki City, Gifu 501-3802, Japan; 4 Department of Respirology, Gifu University Graduate School of Medicine, Yanagido 1-1, Gifu 501-1194, Japan

**Keywords:** Stereotactic body radiotherapy, Non-small cell lung cancer, Elderly patients, Radiation pneumonitis

## Abstract

**Background:**

Stereotactic body radiotherapy (SBRT) for non-small cell lung cancer (NSCLC) is primarily a treatment option for medically inoperable patients, who are often elderly. However, few studies report the effects of SBRT in elderly patients. Thus, we retrospectively analyzed clinical outcomes and feasibility following treatment of very elderly patients (age ≥ 85 years) with stage Ι NSCLC and younger patients (age < 85 years) with SBRT in our institution.

**Methods:**

From January 2006 to December 2012, 81 patients (20 very elderly; median age, 80 years; age range 64–93 years) with stage Ι NSCLC received SBRT. Prescription doses of 48 Gy were delivered in 4 fractions over 2 weeks or doses of 60 Gy were delivered in 10 fractions over 3 weeks.

**Results:**

Local control was achieved in 91.8% of all patients at 3 years (83.1% and 93.8% of very elderly and younger patients, respectively), and the 3-year overall survival (OS) rate was 69.4% (40.7% and 75.0% of very elderly and younger patients, respectively). OS rates were significantly shorter for the very elderly group than for the younger group, with a 3-year cause-specific survival (CSS) rate of 77.9% (50.4% and 81.6% of very elderly and younger patients, respectively) and a 3-year progression-free survival (PFS) rate of 59.5% (44.7% and 63.5% in very elderly and younger groups, respectively). Multivariate analysis revealed a significant correlation between T stage and OS. Grades 2 and 3 radiation pneumonitis (RP) occurred in 7 (8.6%) and 2 (2.5%) patients, respectively. Among patients of very elderly and younger groups, grade 2 RP occurred in 4 (20%) and 3 (4.9%) patients, and grade 3 occurred in 2 (10%) and 0 (0%) patients, respectively. No grade 4 or 5 toxicity was observed, RP was significantly more severe among very elderly patients.

**Conclusions:**

SBRT for stage Ι NSCLC was well tolerated and feasible in very elderly patients. The efficacy of SBRT was comparable to that achieved in younger patients, although very elderly patients experienced significantly more severe RP. Although this study cohort included only 20 very elderly patients, the present data suggest that decreasing volumes of normal lung tissues exposed to ≥ 20 Gy and mean lung doses reduces the risk of RP in very elderly patients. The present data warrant studies of larger very elderly cohorts.

## Background

Numbers of elderly patients with non-small cell lung cancer (NSCLC) are currently increasing [1]. However, these patients are less likely to receive surgical resection due to comorbid conditions, higher intraoperative risks, and personal preference to avoid definitive surgery. Radiotherapy offers a curative alternative for elderly patients with NSCLC, although conventional radiotherapy is not curative [2]. Silbley GS et al. showed that higher than conventional doses of radiotherapy improved survival in patients with medically inoperable stage I NSCLC [3]. Furthermore, stereotactic body radiotherapy (SBRT) presents a promising treatment for patients with stage I NSCLC who are medically inoperable or refuse surgery, with improved efficacy and lower complication rates [4-7]. Previously, the effectiveness of lobectomy, sublobar resection, conventional radiotherapy, SBRT, and observation based treatment strategies were compared with conventional radiotherapy in elderly patients. In this study, overall survival (OS) was significantly improved following SBRT and was similar to that after lobectomy [8]. Other recent reports also indicate that SBRT is an effective treatment option for the elderly (age ≥ 75 years), with minimal toxicity [9-12] and similar OS outcomes to those achieved with surgery [13]. According to reports from Japanese institutions, SBRT is primarily performed in medically inoperable NSCLC patients with median ages of 76–78 years [14-17]. Japan has one of the world’s fastest aging societies, with a mean life expectancy at birth of 83 years in 2011 (79 years for men and 86 years for women) [18]. However, few studies report outcomes of SBRT in elderly patients with NSCLC. Thus, in the present study, we retrospectively analyzed clinical outcomes and feasibility of SBRT in 20 very elderly patients (≥85 years) with stage Ι NSCLC who exceeded the Japanese life expectancy at birth, and made comparisons with NSCLC patients of < 85 years.

## Methods

### Eligibility criteria

Eligibility criteria were as follows: (1) identification of T1N0M0 or T2aN0M0 (stage Ι) primary lung cancer according to the Union for International Cancer Control in the 7th lung cancer TNM classification and staging system using computed tomography (CT) of the chest and upper abdomen, bone scintigraphy, and brain magnetic resonance imaging, (2) confirmation of NSCLC from histology or clinical information such as increased maximum standardized uptake valued (SUVmax) on 18-fluoro-deoxyglucose-positron emission tomography (FDG-PET), tumor enlargement on CT images, or elevated tumor marker levels during the observation period, (3) predominantly peripheral localization of the tumor, and (4) arterial oxygen pressure of ≥ 60 mmHg and predicted postoperative forced expiratory volume of ≥ 700 ml at 1 s. These respiratory criteria are identical to those prescribed by the Japan Clinical Oncology Group 0403 [19]. Patients with medicated interstitial pneumonia or a history of radiotherapy to the chest and lungs were excluded, whereas age was not considered a contraindication. Medical operability of tumors was assessed by a multidisciplinary board. Our institutional Medical Ethics Committee approved the treatment protocol, and all patients submitted written informed consent before inclusion in the study.

### Patient characteristics

From January 2006 to December 2012, 81 patients with stage Ι NSCLC received SBRT. Table 1 summarizes the pretreatment characteristics of the 81 patients, who were divided into age groups of very elderly (≥85 years; n = 20, 24.7%) and younger (<85 years; n = 61, 75.3%) patients. The median age of all patients was 80 years (range, 64–93; 3 over 90 years), and 64 were male (79%) and 17 were female (21%). Very elderly patients included 11 females and 9 males. Of the 17 patients for whom histological diagnosis of NSCLC was not possible, 13 were very elderly. Among all patients, 61 (75%) were assessed as inoperable, and only 4 very elderly patients were considered medically operable.

**Table 1:** Patient and tumor characteristics by age group (very elderly and younger status)

	**All**	**Very elderly ≥ 85 years**	**Younger < 85 years**	***p *** **value**
	**(n = 81)**	**(n = 20)**	**(n = 61)**	
Age (years)				
Median (range)	80 (64–93)	86 (85–93)	78 (64–84)	
Gender				0.002*
Female	17	9	8	
Male	64	11	53	
Performance status (ECOG)				0.44
0/1/2/3/4	55/24/2/0/0	14/6/0/0/0	41/18/2/0/0	
T stage				0.57
T1a/T1b/T2a	42/21/18	9/7/4	33/14/14	
Histology				0.62
Adenocarcinoma	35	11	24	
Squamous cell carcinoma	27	5	22	
Unclassified NSCLC	2	0	2	
Unproven	17	4	13	
Tumor location				0.64
Central/Peripheral	6/75	1/19	5/56	
Tumor opacity				0.41
Solid/GGO	79/2	20/0	59/2	
Operability				0.49
Operable/Inoperable	21/60	4/16	17/44	
Breath-hold				0.02*
Yes/No	50/31	8/12	42/19	
Total dose				0.49
48 Gy/60 Gy	60/21	16/4	44/17	
CTV (cc)				0.92
Mean ± SD (range)	19.5 ± 18.5 (1.1–91)	19.1 ± 19.3 (2.5–68)	19.7 ± 18.4 (1.1–91)	
PTV (cc)				0.46
Mean ± SD (range)	69.1 ± 49.7 (9.3–224)	76.3 ± 65.4 (14.3–224)	66.8 ± 43.8 (9.3–205)	
V20 (%)				0.64
Mean ± SD (range)	5.9 ± 3.2 (1.5–16)	6.2 ± 3.3 (1.6–14.9)	5.8 ± 3.2 (1.5–16)	
MLD (Gy)				0.30
Mean ± SD (range)	3.9 ± 1.6 (1.2–9.2)	4.2 ± 1.5 (1.6–7.6)	3.8 ± 1.6 (1.2–9.2)	

Abbreviations: *ECOG* Eastern Cooperative Oncology Group, *NSCLC* non-small cell lung cancer, *GGO* ground-glass opacity, *CTV* clinical target volumes, *PTV* planning target volumes, *V20* volumes of normal lung tissue exposed to **≥** 20 Gy, *MLD* mean lung doses.

### Treatment methods

SBRT was performed with 6 MV X-rays using a CLINAC C21EX linear accelerator (2006–2009; Varian Medical Systems, Palo Alto, CA, USA) or a Novalis Tx linear accelerator (2010–2012; BrainLAB, AG, Germany). A CT simulator and a 3D radiotherapy planning system (ECLIPSE, Version 6.5, 7.5; Varian Medical Systems) were used to plan treatments for all patients. A BlueBAG system (Medical Intelligence, Munich, Germany) was used to immobilize patients. To maintain tumor positions during irradiation, patients were instructed in the self-controlled breath-hold technique using an Abches (APEX Medical, Tokyo, Japan) respiratory monitoring system [20]. This system was used during CT scanning for treatment planning and irradiation, and breath was held at inspiration or expiration. CT data sets comprised 3 scans for each patient, with a slice thickness of 2.5 mm. For patients who were unable to hold their breath long enough, irradiation was performed and CT images for treatment planning were obtained under normal breathing during both expiratory and inspiratory phases. Data sets were combined, and gross tumor volumes (GTV) were contoured for each patient. Clinical target volumes (CTV) were equal to GTV and the internal target volume (ITV) was the sum of CTV. Planning target volumes (PTV) were determined by adding 3- to 5-mm margins around the ITV, with a leaf margin of 5 mm. Prescription doses of 48 Gy were predominantly delivered in four fractions at the isocenter using 8–11 conformal static ports, and patients were treated biweekly. For tumors located centrally or adjacent to critical organs with large CTV, the prescribed dose was a total of 60 Gy in 10 fractions over 3 weeks. Dose calculations were performed using the convolution method, and the Batho power-law method was used to correct for tissue heterogeneities. Dose constraints for organs at risk were maintained on the basis of the criteria described by JCOG 0403 [19,21].

### Evaluation

Follow-up after SBRT comprised CT scans at 1 month, and then at 3-month intervals for the first 2 years. Thereafter, follow-up CT scans were performed every 4 months. Local recurrence was diagnosed according to pathological confirmation, high uptake on FDG-PET (SUVmax ≥ 8) [22,23], enlargement of tumor size, or the presence of a mass-like consolidation shadow with disappearance of air bronchogram [24,25]. Toxicity was evaluated using the Common Terminology Criteria for Adverse Events, version 4.0. Median follow-up was 29.0 months, ranging from 5.0 to 84.0 months (median 22.5 months) in patients aged ≥ 85 years, and was 30.0 months for all patients < 85 years.

### Statistical analysis

Continuous quantitative variables were compared using Student’s *t* test, ordinal quantitative variables were compared using Mann–Whitney *U* test, and qualitative variables were compared using chi-squared test with Fisher’s exact test. The Kaplan–Meier method was used to calculate local control (LC), overall survival (OS), cause-specific survival (CSS), and progression-free survival (PFS) rates, and group comparisons were made using the log-rank test. The Cox proportional hazard model was used to identify predictors of OS in both univariate and multivariate analyses. Multivariate analyses were performed for variables with probability (*p*) values of < 0.20 in univariate analysis, and differences were considered significant when *p* < 0.05. All statistical analyses were performed using StatView software version 5.0 (SAS Institute, Cary, NC, USA).

## Results

### Patient and tumor characteristics

Table 1 summarizes the pretreatment characteristics of 81 patients, and tumors from very elderly (≥85 years) and younger (<85 years) patients. Numbers of females were significantly greater in the very elderly group than in the younger group (*p* = 0.002). Numbers of patients who were capable of self-controlled breath-holding during irradiation were significantly fewer in the very elderly group than in the younger group (*p* = 0.02). CTV were similar in each group, whereas PTV, V20 (the percentage of the normal lung volume, after subtracting PTV following radiation with ≥ 20 Gy), and mean lung doses (MLD) were slightly but insignificantly greater in the very elderly group than in the younger group. No other significant differences were found between the groups.

### Local control and survival

**Figure 1:**
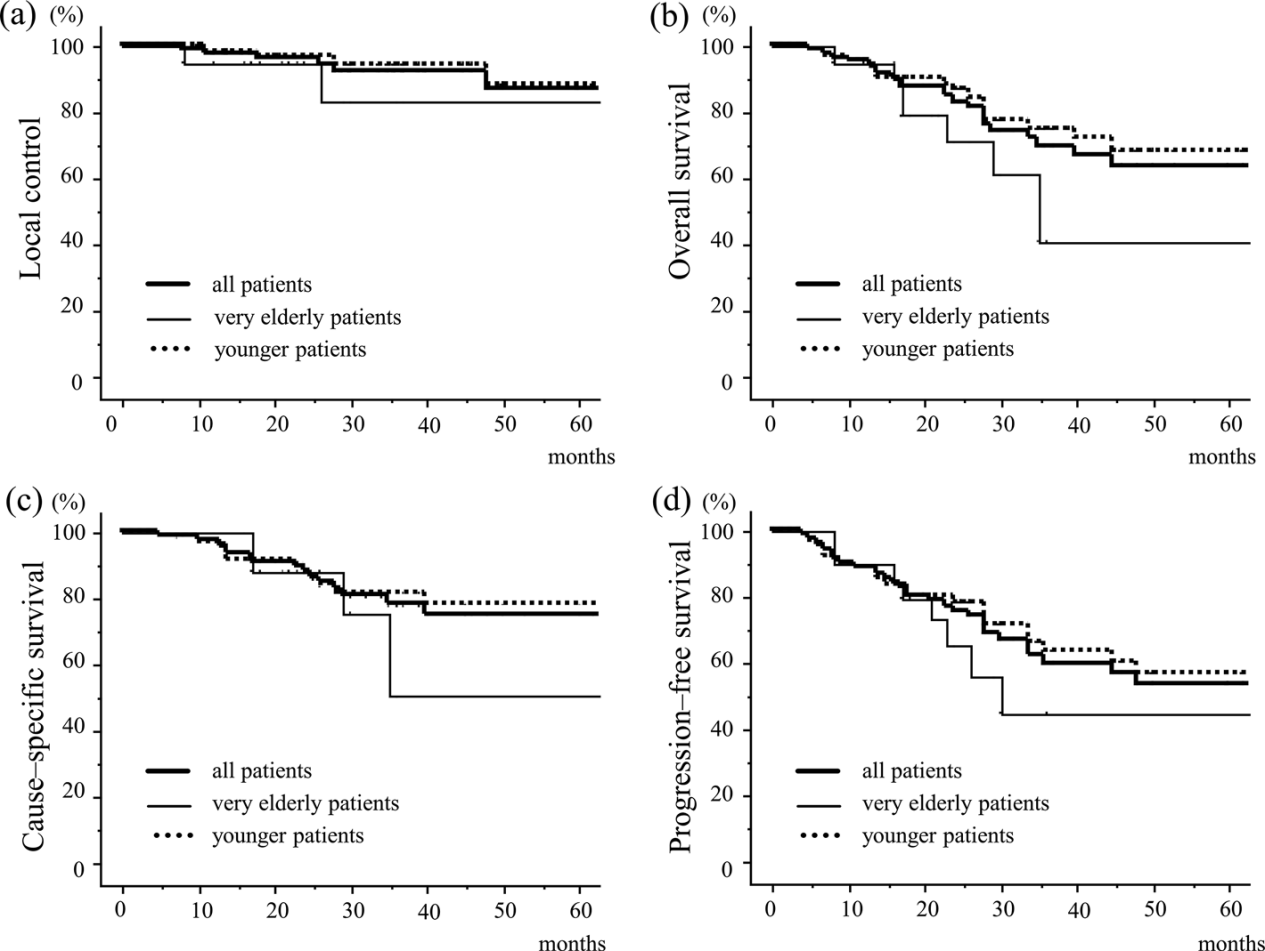
**Local control and survival according to Kaplan–Meier method. (a)** local control (LC) curve, **(b)** overall survival (OS) curve, **(c)** cause-specific survival (CSS) curve, and **(d)** progression-free survival (PFS) curve. Bold solid line, all patients (n = 81); thin solid line, very elderly patients (age ≥ 85 years; n = 20); dotted line, younger patients (age < 85 years; n = 61). Significant differences in OS were found between very elderly and younger groups (*p* = 0.03). There were no significant differences in LC, CSS, or PFS between the groups.

Among all 81 patients, 1-, 2-, and 3-year LC rates were 97.4%, 95.9%, and 91.8%, respectively, and did not differ significantly between very elderly and younger patient groups (95.0%, 95.0%, and 83.1%, and 98.2%, 96.4%, and 93.8% , respectively; Figure 1a and Table 2). Among all patients, 1-, 2-, and 3-year OS rates for all patients were 95.1%, 82.9%, and 69.4%, respectively. These were significantly shorter among very elderly patients (95.0%, 71.2%, and 40.7%, respectively) than in the younger group (95.1%, 86.4%, and 75.0%, respectively; *p* = 0.0306; Figure 1b and Table 2). Among all patients, 1-, 2-, and 3-year CSS rates were 97.5%, 87.8%, and 77.9%, respectively, and did not differ significantly between very elderly (100%, 88.2%, and 50.4%, respectively) and younger patients (96.7%, 87.8%, and 81.6%, respectively; Figure 1c and Table 2). Similarly, 1-, 2-, and 3-year PFS rates for all patients were 88.9%, 75.5%, and 59.5%, and did not differ between very elderly (90.0%, 65.2%, and 44.7%, respectively) and younger patients ( 88.5%, 78.3%, and 63.5%, respectively; Figure 1d and Table 2).

**Table 2:** Local control and survival rates

	**1 year (%)**	**2 years (%)**	**3 years (%)**	***p *** **value**
Local control for all patients	97.4	95.9	91.8	0.32
Very elderly patients (n = 20)	95.9	95	83.1	
Younger patients (n = 61)	98.2	96.4	93.8	
Overall survival for all patients	95.1	82.9	69.4	0.03*
Very elderly patients (n = 20)	95	71.2	40.7	
Younger patients (n = 61)	95.1	86.4	75.0	
Cause-specific survival for all patients	97.5	87.8	77.9	0.42
Very elderly patients (n = 20)	100	88.2	50.4	
Younger patients (n = 61)	96.7	87.8	81.6	
Progression-free survival for all patients	88.9	77.5	59.5	0.11
Very elderly patients (n = 20)	90	65.2	44.7	
Younger patients (n = 61)	88.5	78.3	63.5	
*p < 0.05				

Univariate and multivariate analyses of predictors of OS in all patients are shown in Table 3, and age, gender, performance status, T stage (T1a vs. T1b or T2a), CTV, PTV, histology, tumor location, operability, and total doses were identified as independent variables. However, in univariate analyses, only age, CTV, and T stage were correlated with OS, with *p* values of < 0.20. Because CTV was strongly related to T stage, subsequent multivariate analyses were performed with only age and T stage, and T stage was significantly correlated with OS (*p* = 0.04; Table 4).

**Table 3:** Summary of univariate analyses of overall survival

**Parameters**	**n**	**Hazard ratio (95% CI)**	***p *** **value**
Age		1.05 (0.98–1.12)	0.19*
Gender			
Female	17	1	0.72
Male	64	1.21 (0.52–3.65)	
PS (ECOG)			
0	55	1	0.61
1–2	26	1.23 (0.42–3.65)	
T stage			
T1a	42	1	
T1b, T2a	39	2.38 (1.05–5.39)	0.04*
CTV		1.01 (1.00–1.03)	0.05*
PTV		1.00 (0.99–1.01)	0.29
Histology			0.78
Adenocarcinoma	35	1	
Squamous cell carcinoma	27	1.46 (0.47–4.57)	0.51
Unproven	17	1.18 (0.35–3.92)	0.79
Tumor location			
Peripheral	6	1	
Central	75	1.29 (0.30–5.51)	0.73
Operability			
Yes	21	1	
No	60	1.03 (0.43–2.43)	0.94
Total dose			
48 Gy	60	1	
60 Gy	21	0.99 (0.93–1.07)	0.87

Abbreviations: *ECOG* Eastern Cooperative Oncology Group, *CI*confidence interval.

*p < 0.2.

**Table 4:** Summary of multivariate analysis

**Variables**	**Hazard ratio (95% CI)**	***p *** **value**
Age	1.05 (0.98–1.12)	0.19
T stage (T1a vs. T1b, T2a)	2.41 (1.05–5.50)	0.04*

Abbreviations: *CI* confidence interval.

*p < 0.05.

### Toxicity

SBRT was well tolerated, and all patients completed the scheduled irradiation course without hospitalization. Grades 2 and 3 RP developed in 7 (8.6%) and 2 (2.5%) patients, respectively. In the very elderly and younger groups, grade 2 RP was observed in 4 (20%) and 3 (10%) patients, and grade 3 RP was observed in 2 (10%) and 0 (0%) patients, respectively. No patients suffered from grade 4 or 5 toxicity. However, RP was more severe in the very elderly group than in the younger group (*p* = 0.002; Table 5). In multivariate analyses of RP grade, age, CTV, PTV, V20, and MLD, age was significantly related to the severity of RP (*p* = 0.018), whereas CTV, PTV, V20, and MLD were not. However, V20 and MLD showed predictive tendencies for the severity of RP, with *p* values of 0.054 and 0.052, respectively (Table 6). Late toxicities included CT diagnosed rib fractures in 15 (18.5%) of 81 patients (5 (25%) in the very elderly group and in 10 patients (16.3%) of the younger group. Only 2 patients (2.4%) in the younger group complained of transient chest pain. Rib fracture rates did not differ significantly between age groups (Table 5). Other adverse events included nonmalignant pleural effusion in 5 patients (2 very elderly patients and 3 and younger patients), atelectasis in 3 younger patients, and pneumothorax in 1 younger patient.

**Table 5:** Toxicity and adverse events in very elderly and younger patients

	**All**	**Very elderly**	**Younger patients**	***p *** **value**
	**(n = 81)**	**(n = 20)**	**(n = 61)**	
Radiation pneumonitis				
≥ Grade 2	9 (11.1%)	6 (30%)	3 (4.9%)	0.002*
Grade 2	7 (8.6%)	4 (20%)	3 (4.9%)	
Grade 3	2 (2.5%)	2 (10%)	0	
Rib fracture	15 (18.5%)	5 (25%)	10 (16.3%)	0.41

*p < 0.05.

**Table 6:** Radiation pneumonitis according to grade

	**All**	**Grades 0, 1**	**Grades 2, 3**	***p *** **value**
	**(n = 81)**	**(n = 72)**	**(n = 9)**	
Age (years)				
median (range)	80 (64–93)	80 (64–88)	85 (70–93)	0.018*
CTV (cc)				
median (range)	12.4 (1.1–91)	12.4 (1.1–61.8)	14.8 (2.9–91)	0.49
PTV (cc)				
median (range)	57.0 (9.3–224)	55.9 (9.3–224)	57.6 (22.7–215)	0.43
V20 (%)				
median (range)	5.3(1.5–16)	5.1 (1.5–16)	6.8 (3.7–11.1)	0.054
MLD (Gy)				
median (range)	3.8 (1.2–9.2)	3.75 (1.2–9.2)	4.8 (2.9–6.9)	0.052

*p < 0.05.

## Discussion

Elderly populations are growing in many countries, including Japan. Although lung cancer is a leading cause of death, patients aged ≥ 80 years account for only 14% of all lung cancer patients [26]. Because the life expectancy of Japanese at birth was 83 years in 2011 for both sexes, and because men and women who are 85 years old are expected to live for an additional 6.0 and 8.1 years, respectively, radical treatment should be considered for elderly patients [18]. Surgery is the standard treatment for stage I NSCLC. However, elderly patients are often unsuitable for surgery and prefer non-surgical options. The prognosis for untreated stage I NSCLC is poor, with a median survival period of only 13 months [27]. Radiotherapy is considered a curative alternative for these patients, primarily because reported outcomes of SBRT are similar to those of surgery [28]. Accordingly, SBRT is often the primary treatment option for patients with stage I NSCLC who are medically inoperable or refuse surgery, and Palma et al. [29] proposed SBRT as the standard care for inoperable elderly patients.

The median age of patients receiving SBRT for stage I NSCLC in Japanese institutions is 76–78 years [14-17], but was 80 years in the current study, which may reflect the rural location of our hospital. Significantly more women were aged ≥ 85 years than men, reflecting the well known longer average life span of women than men. In this study, we used a self-controlled breath-hold technique to reduce ITV and PTV. However, patients in the very elderly group could not hold their breath for a sufficient period of time, leading to slightly but insignificantly higher mean PTV, V20, and MLD values in the very elderly group. No other significant differences in patients or tumor characteristics were found between very elderly and younger patient groups.

Several recent studies show that SBRT is an effective treatment, causing only minimal toxicity in elderly patients with NSCLC (Table 7), and leads to excellent 3-year LC rates of 82.3%–100% [9,11,12]. In the present study, LC rates were 83.1% and 93.8% among very elderly and younger patients, respectively, but did not differ significantly between the groups. LC rates for inoperable patients receiving SBRT for stage I NSCLC were reportedly between 83.0% and 97.6% at 3 years [5-7], and this was similar among elderly patients.

**Table 7:** Studies of stereotactic body radiotherapy for stage I NSCLC in the elderly

**Author**	**Age (range)**	**n**	**T stage**	**Doses**	**Local control**	**Overall survival**
Haasbeek CJ et al. [9]	≥ 75 (75–91)	193	T1 118	60 Gy/3 fr	89% at 3 years	45% at 3 years
			T2 85	60 Gy/5 fr		
				60 Gy/8 fr		
Takeda A et al. [12]	≥ 80 (80–91)	109	T1a 32	50 Gy/4 fr	82.3% at 3 years	53.7% at 3 years
			T1b 35	40 Gy/ 5 fr		
			T2 42			
Sandhu AP et al. [11]	≥ 80 (80–89)	24	T1 18	48 Gy/4 fr	100%	74% at 2 years
			T2a 6	56 Gy/5 fr	(4.3–61.2 months)	
This study	≥ 85 (85–93)	20	T1a 9	48 Gy/4 fr	83.1% at 3 years	71.2% at 2 years
(Hayashi S et al.)			T1b 7	60 Gy/10 fr		40.7% at 3 years
			T2a 4			

The OS rate for all 81 patients was 69.4% at 3 years, and 1-, 2-, and 3-year OS rates in the very elderly group were 95.0%, 71.2%, and 40.7%, respectively. Although these rates were significantly lower than in the very elderly group, CSF rates did not differ between the groups. Eight patient deaths occurred in the elderly group during the present observation period. Among these, 4 were due to lung cancer (1 with local failure and distant metastases, 2 were due to distant metastases only, and 1 was due to pleuritis carcinomatosa. The other 4 patients died of unrelated illnesses (2 of heart failure, 1 of cerebral infarction, and 1 of pneumonia that was not related to radiation pneumonitis). Thus, very elderly patients tended to die of causes other than NSCLC.

Prognostic factors for OS that were identified in univariate analysis included age, T stage (T1a vs. others), and CTV. However, in subsequent multivariate analyses, only T stage was a significant prognostic factor for OS, indicating that tumor size is a stronger prognostic factor than age. In agreement, Palma et al. [30] showed that survival after radical treatment (radical radiotherapy or surgery) for stage I NSCLC is dependent on tumor stage but not age. They also suggested that elderly patients should not be excluded from radical treatments based on age alone. Moreover, T stage (T1a vs. T1b or T2a), tumor diameter, and sex were previously reported as significant prognostic factors for NSCLC following SBRT [31,32]. In the present study, prognostic factors were not evaluated in the very elderly due to low patient numbers and limitations of natural life span. Takeda et al. [12] reported predictors of short OS in medically inoperable patients aged ≥ 80 years, including low body mass index, high T stage, and high C-protein level. The present data also show that operability was not a significant prognostic factor. However, because this is assessed by multidisciplinary boards, a bias may exist between institutions. Nonetheless, 4 very elderly operable patients remained alive without recurrence during the observation period (18–36 months). In this study, we did not analyze factors associated with FDG-PET because not all patients were assessed using this procedure. However, we previously reported that pretreatment SUVmax values from FDG-PET or CT were predictive of disease-free survival in SBRT-treated patients with pathologically or cytologically confirmed stage I NSCLC [22]. However, some reports show no relationship between SUVmax and SBRT treatment outcomes for NSCLC [33,34]. Thus, the prognostic value of SUVmax in these patients remains controversial.

Toxicity of SBRT is primarily related to RP. However, whereas grades 2 and 3 RP reportedly occur in 4.6%–13.8% and 0%–20% of patients, respectively [7,9-11,19,2,30,31], reported rates of grade 4 and 5 RP are very low. According to a survey of SBRT in Japan, grade 5 RP occurs in 0.6% of cases and is predominantly associated with interstitial pneumonia [21,35]. Similarly, among the present patients, rates of grades 2 and 3 RP were 8.5% and 2.5%, respectively, and no grade 4 or 5 RP was observed. However, grades 2 and 3 RP were observed in 20% and 10% of very elderly patients, respectively. Severe RP was observed more frequently in the very elderly group than in the younger group. However, no younger patients, and only two very elderly patients, suffered from grade 3 RP, and the severity of RP was significantly correlated with age. The incidence of ≥ grade 2 RP in the very elderly group was greater than that reported in other studies of elderly patients (Table 7). However, previous reports included patients aged ≥ 75 or 80 years, and and did not include data from patients aged ≥ 85 years. Nonetheless, the increased severity of RP among very elderly patients may reflect reduced cardiopulmonary functions and physical conditions. PTV, V20, and MLD are reportedly risk factors for RP in SBRT treated patients with NSCLC [30,36-38]. In the present study, CTV values were similar between the groups, whereas PTV, V20, and MLD values were slightly higher in the very elderly group, and V20 and MLD were almost significant predictors of RP severity (Table 6). Very few of the present elderly patients were able to perform the breath-hold technique sufficiently to maintain tumor position during irradiation and reduce the volume of irradiated healthy lung tissue, which may have increased PTV, V20, and MLD values. Hence, efforts to decrease these values should be prioritized to reduce the risk of RP using methods such as four-dimensional CT with respiratory gating or real-time tumor-tracking radiation therapy systems [31,39].

Rib fracture was observed on follow-up CT in 18.5% of all patients (25% in the very elderly group and 16.3% in the younger group). Only two (2.4%) patients in the younger group complained of chest pain, and no patients exhibited symptoms of grade 3 or more. All elderly patients with rib fractures were asymptomatic, and no significant differences in rib fracture rates were found between the groups. In a study by Nambu et al. [40], rib fractures occurred in 23.2% of patients, and pain to the chest wall was reported in 10.2% of patients. The rib fracture rate in the current study was much higher than that reported in other studies of elderly patients, but was similar to that reported by Nambu et al. [40]. Potentially, the follow-up period, tumor locations, and parameters of CT scanning may have increased rib fracture rates in the present study. Previous studies showed that female gender, lateral tumor location, small tumor-chest distance, doses to 8 cc of the chest wall, and doses to 2 cm^3^ of the rib were significant prognostic factors for rib fracture [41-43], However, age was not a risk factor for rib fracture in the present study, and symptoms were generally mild or asymptomatic if present. Thus, rib fracture is not a major concern during treatment of very elderly patients with SBRT.

## Conclusion

SBRT for very elderly patients (age ≥ 85 years) with stage Ι NSCLC was well tolerated and feasible, with comparable efficacy to that observed in younger patients. Although very elderly patients suffered significantly more severe RP than younger patients, SBRT decreased V20 and MLD and reduced the risk of RP in very elderly patients. Although only 20 very elderly patients with NSCLC were included, the present analyses indicate that SBRT is curative, and warrant future studies with larger patient numbers.

## Competing interests

The authors declare that they have no competing interests.

## Authors’ contributions

All authors read and approved the final manuscript. SH contributed to the study design, data collection, and analysis, and drafted the manuscript. HT contributed to the study design, data collection and analysis, and approved the final version of the manuscript. YK participated in data analysis and interpretation. YO performed clinical evaluations of patients at follow-up and collected data. HH contributed to study data abstraction, critical reviewed and improved intellectual content, and approved the final version of the manuscript.
